# Co-Creation with Older Adults to Improve User-Experience of a Smartphone Self-Test Application to Assess Balance Function

**DOI:** 10.3390/ijerph17113768

**Published:** 2020-05-26

**Authors:** Linda Mansson, Maria Wiklund, Fredrik Öhberg, Karin Danielsson, Marlene Sandlund

**Affiliations:** 1 Department of Community Medicine and Rehabilitation, Section of Physiotherapy, Umeå University, 901 87 Umeå, Sweden; maria.e.wiklund@umu.se (M.W.); marlene.sandlund@umu.se (M.S.); 2 Department of Radiation Science, Umeå University, 901 87 Umeå, Sweden; fredrik.ohberg@umu.se; 3 Department of Informatics, Umeå University, 901 87 Umeå, Sweden; karin.danielsson@umu.se

**Keywords:** aged, mHealth, mobile application, self-assessment, community-based participatory research, muscle strength, postural balance, qualitative research, accidental falls, UX Honeycomb model

## Abstract

This co-creation study aimed to develop a smartphone self-test application for balance and leg strength in collaboration between older adults and the research team. The paper describes older participants’ preferences for, and their contribution to, the application design. Technology to assess movements is available in smartphones with built-in sensors, and one of the challenges is to develop a valuable self-test for older adults. The participants contributed to the design of the application’s instructions and user interface. Multiple data collection methods were used: user-test with Think aloud method, mock-ups, homework assignment as co-researcher, audio and video recordings. Qualitative content analysis with a deductive-inductive approach was used, guided by the Optimized Honeycomb model for user experience (UX) as a categorization matrix. The analysis resulted in 17 subcategories within the seven facets of the UX Honeycomb model (findable, accessible, usable, desirable, credible, useful, and valuable), and describes the older participants’ preferences and experiences. The main results were participants’ desire to know why, to get clear and appropriate information, and expectations of the self-test to be useful. It was feasible and valuable to develop the self-test application in co-creation with the intended user-group, in order to get direct feedback and suggestions for the development.

## 1. Introduction

The aging population is increasing rapidly, with the number of people over 60 years old estimated to reach two billion in 2050 [1]. With higher age the risk of accidental falls also increases, which is a major problem around the world [2]. The greatest number of fatal falls occurs in people 65 years and older [2], and strength, balance and gait impairments are risk factors for falling [3]. Consequently, new strategies are needed to prevent falls, both within and outside the healthcare system. Various health technologies can be used as important strategies in health promotion and prevention. A review study about the use of smartphones in health promotion included six studies regarding fall prevention or fall detection, already in 2013 [4].

In fall prevention, the use of smartphone applications offers promising tools to assess, monitor, and support exercise to strengthen physical functions such as balance. This is in line with the growing use of mobile technology, now also among older adults. Recent statistics from the 28 European union countries, show that 52% of individuals aged 55–74 years use mobile devices to access the internet away from work or home [5]. This has doubled during the last five years for this age group. In Sweden, data from a population survey about internet use presents that 72% in the age group ≥76 years used a smartphone to access the internet and 51% reported doing so daily, while the younger seniors, 66–75 years old, 88% access the internet with a smartphone, and 73% reported daily use [6]. 

Thus, the use of technology is opening up new opportunities for health promotion [7,8]. A state of the art review depicted the area of fall prevention intervention technologies, reporting about the use of smartphones to enable older adults to self-assess and self-manage fall prevention [9]. Research on fall prevention applications is increasing and examples of such applications are the exercise interventions ActiveLifestyle [10] and Standing Tall [11]. Nevertheless, fall prevention assessment by using smartphone technology, is still a novel field. A systematic review outlined a promising potential for using smartphones in balance and fall risk assessments, but further research is needed as many of the applications studied infrequently reported data for validity and/or reliability [12]. Moreover, research on self-assessment using smartphone technology is particularly scarce. Regarding self-assessment applications, positive experiences have been reported among older adults in two user-test studies of fall risk assessment [13,14]. Yet, none of the applications consolidated the self-assessment with sensor measurements from the smartphone. In both studies the self-assessment was based on a balance test categorized as pass or fail by the individual, in combination with questions about, for example, their balance confidence and previous falls. Although portable motion sensors have previously been used to detect human movements, and to successfully measure both balance and leg strength in various studies [12,15,16], sensors have not yet been evaluated for self-test applications. Self-assessments with portable sensors could potentially give older adults an option to assess balance and strength at home, without interaction with health care professionals [16]. Taken together, the expansive use of smartphones opens up the possibilities to create suitable self-test applications for older adults. 

### User Experience and Co-Creation in Application Design

For application design, user experience (UX) is essential. UX has been described as the user’s subjective, situated, complex, and dynamic experience [17]. UX is a combination of aspects of experience, e.g., emotions and desires that goes beyond the more traditional human computer interaction’s instrumental aspect of applications [17]. The UX Honeycomb model by Morville, with its seven facets of usability: useful, usable, desirable, findable, accessible, credible and valuable, is utilized to provide UX perspective in application design [18]. This model is frequently used in applied design work [19]. However, the UX Honeycomb model is sparsely used in scientific research. Some examples from evaluations of different medical related applications have been found, where the UX Honeycomb model was used to analyze UX in relation to their developments of applications or decision supports [20,21,22,23]. Several modifications have been made to the original UX Honeycomb model and one reorganization (the Optimized Honeycomb model) was made to illustrate the practical aspects of the model, introducing how users interact with a product by thinking, feeling, and using it [24]. 

When developing a self-test application it is essential to accommodate for the older adult user, since this age group may have certain additional requirements. However, the user experience still needs to be part of the development. Some previous research of user-tests with older adults, have created different guidelines on how to improve usability and acceptance among older users [25,26,27]. These guidelines describe various aspects from structure of content and how navigable the application is, to factors influencing acceptance of technology. Recommendations from guidelines nevertheless must be tailored to each specific product and might be enhanced by involving the older adult in the development process. When designing an application it is important to find out the needs of the older adults, to facilitate its use. The end-users’ understanding of a self-test is fundamental, especially the design of self-test applications since the correct handling of the test is crucial. 

Co-creation is one way to facilitate correct use and bridge the gap between developers and end-users. The terminology co-creation, co-design, or participatory design, all correspond to a process where the end-user is engaged in the development or design of a product. Both co-creation [28] and participatory design [29] deal with the mutual learning and mixed roles of designer and user when working together in a creative way [28,29]. The co-creation collaborative knowledge is described as a useful link between researchers and the community to gain research impact [30]. To obtain a positive outcome, planning and conduction of the co-creation is vital. Some key principles for co-creation in public health interventions have been reported to include framing the aim, careful sampling, manifesting ownership, defining the procedure, and structured evaluation and reporting [31]. 

To our knowledge no study has published results from a self-test with sensor measurements using smartphone technology to self-assess balance and leg strength in older adults. It is desirable for a self-test to be easy to use, to ensure that the instructions can be followed and to be safe at the same time. The aim of this co-creation study was to, according to the Optimized Honeycomb model for user experience, describe the older adult participants’ preferences for, and their contribution to, the design of the instructions and user interface of a smartphone self-test for balance and leg strength. By using co-creation, we expect that the UX could be reinforced and also give an opportunity for user-tests during the development.

## 2. Materials and Methods

### 2.1. Study Design

An interdisciplinary research team conducted this qualitative co-creation study in collaboration with 10 older adults (≥70 years) who, in the design process, represented intended end-users of the evolving mobile application MyBalance. The study had an iterative design process, which is often used in software development and informatics. The iterative process builds on cyclic work with prototypes, using continuous evaluations and improvements [32]. We define our co-creation process as an interaction with participants during sessions where prototypes were elaborated on. Suggestions from each session led to developments of the application’s user interface and instructions, which were further processed at the subsequent session. Before the co-creation sessions commenced a plan for the five sessions was outlined. The outline was revised after each session due to the co-creation process, depending upon the issues that were brought up. Furthermore, an early prototype of the content (e.g., the videos instructions and interface) was made, in order to have some material to work with from the beginning.

### 2.2. Participants

A purposeful sample of older adults, a mix of men and women, with experience of participating in an earlier fall prevention exercise study was recruited [33]. We aimed to involve participants who, in the previous study had openly shared their opinions, this to obtain candid suggestions during the design process. Some previous experience of using either a computer, tablet, or smartphone was also required. A shortlist of 15 individuals was made and they were contacted by e-mail, with the goal of reaching a suitable group size of 10–12 participants. For user-tests, five people are considered to be sufficient to reveal the majority of usability problems [34]. However, we included a larger group so as to acquire a variety of feedback during the sessions, and to cover for potential non-attendance. Twelve people showed interest, but two revoked participation before co-creation sessions commenced due to time-constraints. Six women and four men, with the mean age 76 ± 3 years, formed the group. Attendance at the sessions varied, two participants attended all sessions and three others attended only two sessions. Due to illness and family issues, two participants had to cancel their attendance for the two final sessions. 

The interdisciplinary research team for the application development consisted of two engineers and three physiotherapists, whereas only the physiotherapists took part in the sessions. Three of the authors (LM, MS, MW) are physiotherapy researchers and were involved in the planning and realization of the sessions. Two participated in all sessions (LM, MS) where their role varied between facilitator and observer during the session, one participated partly in three of the sessions (MW), and one additional physiotherapist (also senior researcher) attended during one practical workshop for user-tests.

### 2.3. Prototype

A smartphone application with two different test procedures, was predefined by the research team. The self-test included a three times sit-to-stand test (functional leg strength test) and a 30 s static standing balance test with two different foot positions. During the test procedure the smartphone was placed on the lower back, achieved by tying it around the waist with a scarf or other material available to the individual in their home. The idea was to provide short videos with the information on how to perform the self-test and how to use the application. At the first session, so as to have some material to start with, version 1 of the test instruction videos was prepared, as well as an early prototype of the application for the sensor measurements. Parallel inhouse development of the mobile application and algorithms was done by the research team using Android Studio and a Sony Xperia smartphone. Measurement data from a built-in gyroscope and accelerometers was used in the application.

### 2.4. Data Collection during Co-Creation Process

**Figure 1:**
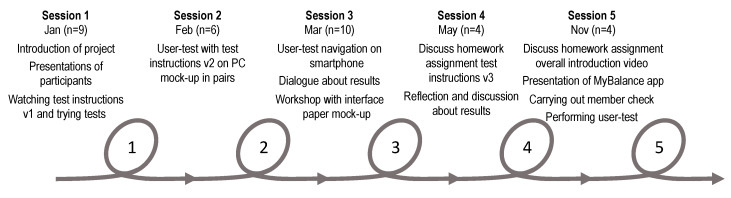
An overview of the design process with tasks from the five co-creation sessions, containing information about each session with number of participants.

The design process is illustrated in Figure 1 with an overview of the five co-creation sessions throughout 11 months. Various procedures were used to collect data on participants’ preferences and suggestions for the design: practical workshop sessions, user-tests, and group discussions. Every session concluded with a whole group discussion to summarize the thoughts and considerations from smaller group practical workshop sessions. Multiple data collection methods were applied, further explained below. 

Session 1 included a short introduction of the project MyBalance and a presentation of the participants, as it was vital for the group dynamics for them to know each other when sharing opinions and ideas during the sessions. The main task of the session was to watch video instructions (v1) on an iPad and try out the two tests (sit-to-stand and standing balance in two positions) in three smaller groups. Each group was given a guide with questions to facilitate the exploration. The practical workshop was done in groups and LM and MS moved between groups to listen in to the conversations and observed how they handled the tasks. In addition, the groups were video recorded. Audio recordings were made both in the small group sessions and in the whole group discussion.

At Session 2 the main task was to navigate a PC mock-up prototype (a PowerPoint model used for showing features of the application) to try the video instructions (v2) in pairs. Both the ability to navigate the MyBalance app and the performance of the tests were observed. The participants’ role was to perform the test or be an active observer; the roles switched after the first test. The researchers’ role was to be a facilitator at the practical workshop, and to video record the user-test. The researcher followed a script in order to give equal instructions in all groups. At Sessions 2 and 3, during user-tests, the Think aloud method [35] was applied. The Think aloud method encourages participants to talk out loud while performing tasks to express what comes into their minds, for example, what they are looking at, or what they are thinking, doing, and feeling. This method gives the observer insight into the participant’s reasoning and facilitates the design process. Audio recordings were made both in the small group sessions and in the following whole group discussion.

Session 3 comprised three practical workshops with different tasks: (1) navigation of the smartphone, with assignments to get familiar with the app, calibrate sensors, enter weight, and try both tests, (2) dialogue about how results of the test performance could be presented in the app, and (3) discussions about the interface with a paper mock-up. All groups did the user-test with the navigation task and a further two groups had a dialogue about results. One group worked with the graphical user interface (paper mock-up), where photographs were also used to document the result. Audio recordings were made both in the small group sessions and in whole group discussions.

Before Session 4 the participants were given a homework task. The task was, as co-researcher collect information by showing the test-instruction videos (v3) to a friend or family member not previously involved in the design process, to find out if instructions were clear enough to perform the self-test independently. Participants got access to the video via a link to a private YouTube channel, and a written guide was supplied with instructions on how to conduct the observation, as well as questions to discuss with the friend or family member. These questions were later used as a basis for discussing the homework task within the whole group at Session 4. Further, the discussions from the previous session on presentation of test results were followed up. Audio recordings were made.

Session 5 included a conversation about a new homework task regarding the overall introduction video. This homework task had the same aim and was done under the same conditions, as the previous one. Moreover, a member check was held to sum up the developments of the MyBalance app during the co-creation process. Finally, video recorded user-tests with the final prototype were done, followed by a short user experience questionnaire. The user-test was completed by three participants as one had to leave the session earlier. Audio recordings were made.

### 2.5. Data Analysis

A deductive-inductive approach to qualitative content analysis [36,37,38] was used to analyze the participants’ preferences for, and their contribution to, the design of the smartphone self-test. As a categorization matrix, we used the Optimized Honeycomb model for user experience [24] (as described below).

#### 2.5.1. The Honeycomb Model

**Figure 2:**
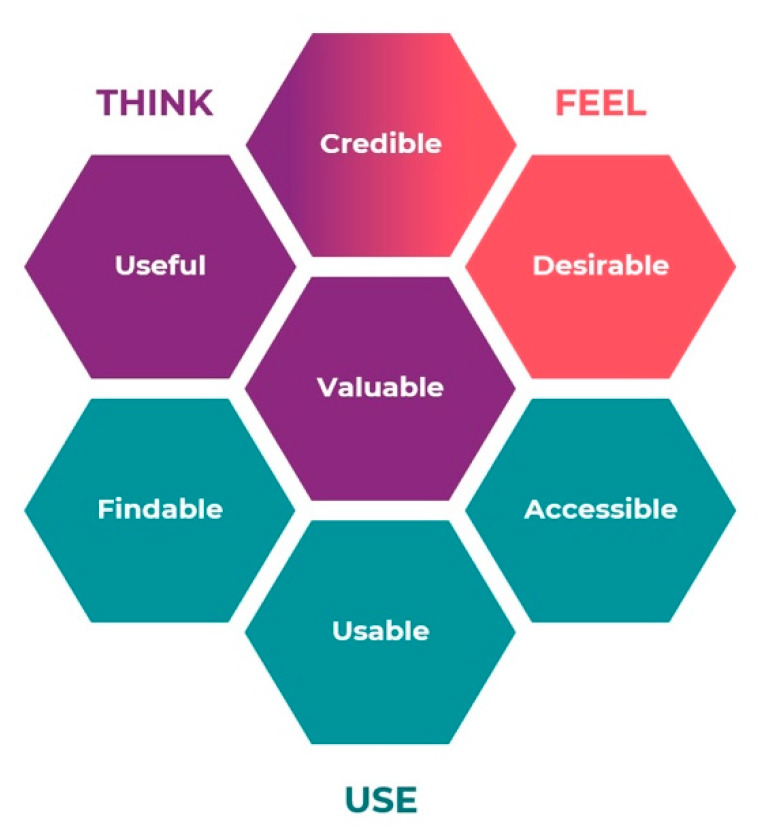
The Optimized Honeycomb model by Karagianni, 2016, used as the categorization matrix for the deductive analysis phase in this study. (Reprinted with permission from the creators).

The deductive part of the qualitative content analysis was performed by using the Optimized Honeycomb model by Karagianni [24]. The model was chosen due to its applicability to analyze the user experience perspective during the design of the self-test application. Moreover, the original UX Honeycomb model by Morville [18] is well known and often used in human–computer interaction [19]. Small amendments have been made to the original model in order to raise its practical use [24]. The facets were grouped into three parts, reasoning about how the user “Feel, Think and Use” the product by making a connection between the seven facets. To facilitate comprehension of the Optimized model, it was reorganized and color coded to visualize the relation between the facets [24]. In Figure 2, Karagianni’s Optimized Honeycomb model is shown. In another re-design, the seven facets were organized as a “UX Staircase” to simplify how the UX Honeycomb model could be followed step by step [39]. We applied this staircase model for visualization of the results. The seven facets are described below, with regard to the original description [18], and our definition in this study:

*Findable*, navigable and easy to find information. In our study this is defined as information in the application being easy to localize.

*Accessible*, manageable for people with disabilities or reduced function. In our study this is recognized as the possibility to access information in the application regarding both physical and cognitive function, as well as accessibility to the actual device, in this case a smartphone.

*Usable*, ability to be used. In our study this is defined as how information is perceived, the feeling of being able to use the information given in the application and being able to perform the tests.

*Desirable*, features with an emotional engagement. In our study this is identified as the group’s needs and interests, how the application could be something to talk about and something everyone wants to use.

*Credible*, influence of elements for users’ trust and belief in the product. In our study this is defined by the confidence in the application, if it feels safe to use, and if it is trustworthy.

*Useful*, to make innovative solutions that are beneficial. In our study this is identified as the application as a whole must fulfil a need.

*Valuable*, deliver value or advance the mission. In our study we defined valuable as the sum of all six categories surrounding it, offering value to the user.

#### 2.5.2. Analytical Procedure

A deductive-inductive approach to qualitative content analysis was used [36,37,38]. The audio recordings were transcribed verbatim, and were, together with the video recorded observations, managed in the analytic software ATLAS.ti (Scientific Software Development GmbH), documenting both verbalized and not verbalized activities. The software was used for the following steps 2 and 3 in the analysis process. Before the analysis started the whole group of authors discussed the model and decided to apply the model’s facets as categories in the deductive analysis phase, and agreed on the above definitions for each category in our study. The subsequent steps were as follows: (1) naïve reading/understanding, (2) identifying meaning units and video sequences according to the aim, (3) condensing text or verbalizing video sequences, (4) deductively sorting the condensed meaning units according to facets of the Optimized Honeycomb model, (5) coding of the condensed meaning units, (6) inductively sorting the codes to outline and label the subcategories within each category. 

Aspects of credibility, dependability, conformability, transferability, and authenticity were addressed throughout the project in order to strengthen the study’s trustworthiness [40]. Multiple data collection methods were chosen to provide rich data to strengthen the trustworthiness. The credibility of the results was considered by having a group of 10 participants in order to get a variety of opinions, and also by describing the researcher role during the analysis. Representativeness, as part of credibility, was addressed by the researchers’ participation and perspectives in the co-creation process, along with the member-check with the participants at the final session. The documented preparations before each session, as well as researchers notes during, and reflective gathering after each session, complemented the data collection from the sessions to enhance dependability. To strengthen conformability, the initial coding and analysis were conducted by the first author (LM), and triangulated together with all four co-authors (FÖ, KD, MS, MW) with different competences and perspectives (e.g., eHealth, engineering, fall prevention, informatics, physiotherapy). To reinforce transferability the preparation phase with the selection of participants, as well as the analysis process and analytical framework, is thoroughly described. The authenticity of the research was established by describing the whole process of this study [40]. Further, the use of quotes in the results, distinguishable to participant and session, is assumed to reinforce credibility in the results by describing the diversity in the material [36]. 

### 2.6. Ethical Considerations

The co-creation study has an approved Ethics application (Umeå Dnr 2017/317-31). Both written and verbal information about the study was provided and all participants signed a written consent form. Participation in five co-creation sessions had limited risks causing ethical concerns, but as the participants shared information about their opinions and personal experiences it was important to ensure that they felt safe within the group and could openly express their ideas. All participants had previously been involved in a fall prevention exercise study and had volunteered to partake in further research projects [33].

## 3. Results

### 3.1. Analysis of Co-Creation Sessions

**Figure 3:**
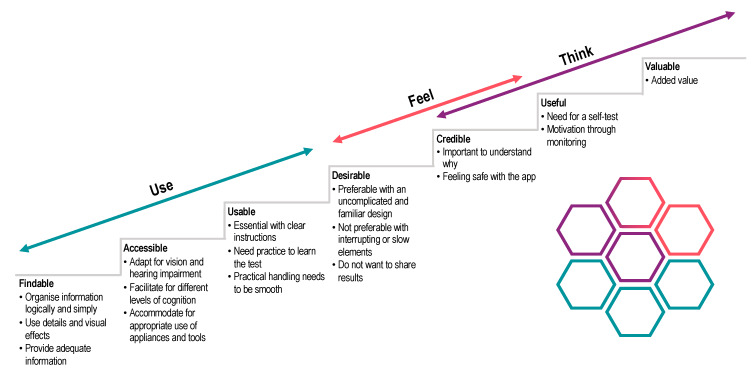
The user experience (UX) from the co-creation design process of the MyBalance app visualized as categories and subcategories in the UX Staircase model.

The analysis was completed using the Optimized Honeycomb model with the seven facets (findable, accessible, usable, desirable, credible, useful, and valuable) as predefined categories in the deductive analysis phase. Within each category the subcategories were inductively generated. In total 17 subcategories, reflecting the participants’ expressed opinions and suggestions, were derived from the five co-creation sessions. See Figure 3 for results in the UX Staircase with our subcategories.

#### 3.1.1. Findable 

The category *findable* relates to participants’ discussions about simplicity and how to facilitate navigation. This category is obtained from data within all sessions.

The participants’ necessity for a clear and simple design formed the subcategory *Organize information logically and simply.* During the practical workshops exploring the user interface, changes such as removing symbols from the heading, and avoiding excessively use of symbols were suggested, so as to improve organization of the interface.

“*If there should be only one symbol, then it is better with the one with the feet together, as you start with that one.*”

Woman 5, 71 years, Session 3

In general, the participants found navigation in the app logical after just some practice, but they sometimes needed guidance for going back or to exit. The use of chapters was suggested to facilitate repetition of certain sections. Another idea for simplification was to provide details, not directly necessary for performing the test, as bonus material for the user who wants more in-depth information. Additionally, they found it confusing when something happened in the app without any notification, for example when the calibration process finished and just returned to start without any confirmation.

The subcategory *Use details and visual effects* concerns how to catch the user’s attention, and how to use colors and contrasts to highlight or to zoom in on important details. The use of bullet points to facilitate navigation was another idea. It was considered helpful to use some symbols and colors in the interface, for example green or arrow up for good results, and red or arrow down for caution or a decline in the results. 

Participants’ need for just the precise amount of information is reflected in the subcategory *Provide adequate information.* Neither too much nor too little information was preferred in order to be able to use the app correctly.

“*I understand that it is a trade-off between whether it becomes too extensive or clear …. that, both to get it clear and not too comprehensive, and you want to have as much as possible in there.*”

Woman 3, 73 years, Session 4

Another suggestion to improve findability for people with hearing impairments was to provide written instructions in addition to the videos. A conclusion from the homework sessions was that the instructions were both findable and perceived uncomplicated even for people not previously involved. 

#### 3.1.2. Accessible

The category *accessible* describes the participants’ perceptions of the ability to access content in the application, which relate to both the information provided and access to the actual device when using the self-test. Most of the information in this category is extracted from the last three sessions. 

The subcategory *Adapt for vision and hearing impairment* captures topics discussed in relation to impairments that could affect the use of the application. Participants acknowledged that individuals with impaired hearing have different needs, but they agreed that spoken instructions, in combination with signals, were considered clear, even for individuals with hearing impairment.

“*But it is very informative and simple. You talk clearly and slowly. My husband is very hard of hearing, and he had no problems to hear.*”

Woman 5, 71 years, Session 5

Distinct preferences were expressed concerning the use of verbal cues or sound signals to indicate change of position during the test performance. These cues and signals were elaborated on in different versions of the application, and finally a preference for a combination of short cues, and signals, was agreed upon. Further, it was considered helpful to make information more accessible by accentuating words and using contrasts of colors in the video instructions.

Participants’ experiences regarding cognitive challenges such as memory, planning, and execution formed the subcategory *Facilitate for different levels of cognition*. It arose from practical workshops where participants followed the test instructions to perform the test. To be able to grasp the instructions they expressed that videos had to be shortened and focus only on the most important points. Occasional confusion was noticed during the test performance, for example, that it was difficult to remember what the next step was. Based on the user-tests at the final session, it became apparent to participants that a relatively good short-term memory was desirable to be able to use the self-test. 

The subcategory *Accommodate for appropriate use of appliances and tools*, illuminates the participants’ considerations regarding the availability of the application and access to technology. For their own part they were happy to use a self-test with their smartphone, but they brought up that maybe not everyone has access to a smartphone, even though it is more common nowadays. Furthermore, they pointed out that the smartphone, during the actual test performance, was not accessible as it was rolled up in a scarf on the lower back. Participants also recognized the possibility of adapting the application for various individuals with different technology experience. 

#### 3.1.3. Usable

The category *usable* comprises the participants’ perceptions on how to manage the application and perform the test. Most material in this category is extracted from the second session, when the first user-test was performed.

The subcategory *Essential with clear instructions* captures the participants’ wish for distinct and clear instructions. They came up with feasible ideas on how to modify the instructions, for example regarding how to guide the sit-to-stand test by, for example, using short verbal cues with specific words such as *“up/down”*, and *“fast”*. Utmost clarity was important to avoid confusion at test performance.

Participants’ experiences from their first attempts using the application is reflected in the subcategory *Need practice to learn the test*. They described a need to familiarize themselves with the test performance a few times, thereafter it became easy to perform the test. They also thought it was helpful to be able to go back and practice with the instructions again to make sure the test was performed correctly before the first assessment.

“*You have to practice first, to be able to do it without instructions later.*”

Man 1, 76 years, Session 2

The subcategory *Practical handling needs to be smooth,* includes issues regarding how participants perceived handling the self-test, and the user interface was considered relevant at first sight. Managing the smartphone and scarf to fasten it on the lower back was reasonable and easier to do than expected after watching the instructions. A few participants considered the calibration process unclear to start with, so the instruction was changed to show the whole process in one video clip, which improved the matter. Further, during practical workshops some technical problems were experienced (interruptions in sound signals, and not being able to follow on to the next step in test procedure, due to poor Bluetooth connection), which affected the participants’ attention negatively. They therefore expressed that they wanted a self-test that worked without hassle. 

#### 3.1.4. Desirable

The category *desirable* refers to participants’ view on how suitable and attractive they thought the application would be for them as end-users. Preferably, it should be something to feel good about and talk about. The participants noticed both desirable and non-desirable features in the application, which contributed to improvements. Data in this category is extracted mostly from the first and third sessions.

The first subcategory *Preferable with an uncomplicated and familiar design,* focuses on participants’ experiences of the user interface and content. To be desirable, they preferred the video instructions to be concise, preventing them from getting bored or losing track while using the application. They wanted the results section to be uncomplicated. To determine how results could be presented, they shared and compared their previous experiences of using other applications, for example, to monitor physical activity or health related issues. Their suggestions were unanimous: results should be presented as line or bar graphs and show change over time. They wanted results presented with numbers and not by symbols to make it understandable. An optional memo function in the results section, where a personal note could be made, was also desired.

Participants found certain features not desirable and this formed the subcategory *Not preferable with interrupting or slow elements.* Signals and voice prompts had to be changed various times so as not to interrupt the test performance. In relation to the slow pace of instructions in the first version of the app, all participants expressed a solid opinion that: just because you are old you are not daft. They all appreciated the change to the more normal speech pace in later versions and felt less diminished.

“*If you are looking at the entire video then, then I would have got fed up before finishing...I would have turned it off before getting to the end.*”

Female 2, 76 years, Session 1

Participants’ resistance to share results with others created the subcategory *Do not want to share results*. Though one remark was that it would be nice to share with a spouse or close friend, particularly if one has done something very well. The strong and unanimous opinion about not wanting to share results on social media was striking. They felt the information was too personal to share. Additionally, the mistrust in social media was expressed and they saw a difference between generations. They felt they wanted to share less than they thought younger people would do. The group also established that they did not want the self-test to be a competition.

“*May I say one more thing, this is not a competition is it? You can post results from competitions on Facebook, but that’s something different.*”

Woman 2, 76 years, Session 3

#### 3.1.5. Credible

The category *credible* describes the participants’ confidence in the application. Topics brought up here were concerns about the sensor measurements and what they tell, how the tests are performed, and which movements are selected to be part of the self-test. Most data in this category was extracted from the first sessions, but participants questioned the application during the whole process, and asked about why things were done in a certain way. 

Participants’ curiosity and their desire to know why and how, formed the subcategory *Important to understand why*. They expressed that it was easier to understand that you must do things in a certain way when you know why. Further, to trust the measurements, it was considered important to understand what might happen if you do something wrong. 

The subcategory *Feeling safe with the app* describes the feeling of being safe while performing the test. Participants’ positive reactions to safety aspects during the test were often confirmed in the co-creation sessions, for example, standing in a corner made them feel secure.

“*You know, somehow… you know subconsciously…(moves his arms to the sides as though searching support)…that you can get support against the wall.*”

Man 4, 82 years, Session 2 (video)

#### 3.1.6. Useful 

The category *useful* exemplifies participants’ perceptions regarding the usefulness of the application for intended end-users and their need for the self-test. Data in this category is obtained mostly from the third and fourth sessions.

The subcategory *Need for a self-test* expresses the participants’ reflections on how to utilize the test. They thought the test could be an alert if decline in physical function occurs, and something to give guidance on how exercise or inactivity affects one’s balance. For the participants, it was obvious that the app was useful for their age group. Moreover, they brought up the question: who are the intended users of the application, older adults or clinicians? They recognized a need for the application in both settings.

“*I think [about results]—better or worse are based on what I did before. Have I made any improvement, or just got worse, or does it look the same? That is interesting. Or will there be a red flag, now you have… what happened to your balance?*”

Female 5, 71 years, Session 3

The motivational aspect of the self-test was expressed by participants and formed the subcategory *Motivation through monitoring.* Self-monitoring could be achieved through goal setting, registration, and comparison over time. In relation to goal setting it might be used as a motivator, to get back to the same level as before an illness or holidays, to set a realistic goal according to age and health status, or to set a goal to motivate exercise. Although, most participants were generally not interested in comparing with others, some asked for possibilities to relate their measures to normal values. In addition, the use of positive feedback in the application, for example, symbols like a smiley figure or green arrow etc. was considered motivating.

“*I agree with that, that you would like to see how it [results] develops. It is exciting to follow, if it progresses or if you have been lazy. Yes, you need that push. Then it is fun as well to follow the results.*”

Female 6, 74 years, Session 3

#### 3.1.7. Valuable

The final category appears as an overall aspect of the other six facets in the model, and illustrates the *Added value* for the end-user to have an application like MyBalance. Participants’ expressed their experiences with increased balance problems and the fear of falling when getting older, and identified a value of being able to self-monitor balance objectively. The group saw the benefits of being able to compare their performance to a normal value. However, one remark was that if you can compare yourself with a normal value and find out that your balance is good, then you might stop exercising. Additionally, the general value of the application was discussed in relation to the question of whether the intended target group actually are using apps and smartphones. The response in the group was “*yes*”, many have access to technology, but the extent of use varies. The participants felt it was justified to develop an application even though it was recognized that not everyone may have access or interest.

“*But people are so different, some run to the doctor or health center for the slightest thing, others stand it to the worst, so it is very difficult to tell [if MyBalance app will help everyone] but the use of smartwatches and smartphones could be a signal that something is wrong. And it is not always…eh, that the inner feeling will alert you—then it could be beneficial with this type of signal to alert that something is wrong.*”

Male 4, 82 years, Session 4

### 3.2. Application Development during the Co-Creation Process

**Figure 4:**
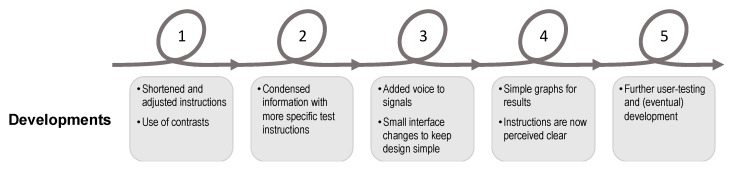
An overview of the design process, describing developments made after each of the five co-creation sessions and consequently further explored in the next iteration.

The developments and improvements of the application after each session are presented in Figure 4. The changes made were in relation to the tasks performed in the five co-creation sessions, as described in Figure 1 in Section 2. Naturally, due to the co-creation process, design changes were made throughout the iterative design process. Changes concerned the interface design, instructions and test procedures, and the most recent version of the prototype was used in each co-creation session. The current version of the smartphone self-test application MyBalance, after the co-creation design process, features three short instruction videos. One video with an overall introduction of the self-test (approx. 5 min) and a separate video for each test procedure (approx. 2 min each). The user interface for both the sit-to-stand test and the static standing balance test have three options: (1) see instruction video, (2) see results from earlier tests, or (3) start the test.

## 4. Discussion

The purpose of this study was to develop a smartphone self-test application, and from the co-creation process describe the participants’ preferences for, and their contributions to, the design of the application. In accordance with the Optimized Honeycomb model, the results can be interpreted from the three parts: Feel, Think, and Use (Figure 3) [24]. Related to the part Feel (categories Desirable and Credible) the participants contributed with the most unanticipated information. They communicated creative ideas and provided perspectives relevant to end-users, the older adults. The suggestions from participants were both applicable and added essential value to the development, regarding how they felt about using the self-test application. In relation to the part Think (categories Credible and Useful), participants’ approval of the purpose of the self-test application was confirmed. Finally, within the part Use (categories Findable, Accessible, and Useable), the interaction with the older adults confirmed much of the practical content, and many suggestions to ease the use of the application evolved during the co-creation process. 

The UX Honeycomb model has to our knowledge been used in three other qualitative studies in medical related research to analyze user-tests and developments, only one of which had an older population. Further, those studies targeted very different areas: online intervention for parents with children with insomnia [23], glaucoma patients using prototype of guidelines about their condition [22], development of Decision Boxes for evidence-based and shared decision-making [20]. Nevertheless, our analysis using the Optimized Honeycomb model, showed findings which resemble previous studies. Within Feel, some noticeable similarities were the users’ desire *to understand why* things are done in a certain way, and their interest in learning new things. The older adults, in our study, had a desire to know the reason behind a particular action to follow instructions and trust the application. Other studies reported knowledge to be empowering for patients with glaucoma [22], and that information reinforced parents to understand the child’s problem [23]. Moreover, in Feel, participants’ wish for an *Uncomplicated and familiar design* was expressed in our study. When the overabundant information existing at the start was removed, and clear and concise instructions provided only the indispensable information, participants were enabled to perform the test correctly. In comparison the importance of using appropriate graphics was stressed in the decision support study [20], and the need for simple graphical aids and the use of color and symbols was expressed by patients with glaucoma [22].

In the part Think, the participants conveyed a *Need for the self-test* application; they wanted to use the application. They expressed both a personal need, and moreover, were very enthusiastic that others would be able to use this app. Other studies described usefulness as participants appreciation of the decision support [20], and dissemination of the guidelines was a key concern for the users in the glaucoma study [22]. Additionally, using results from the self-test was considered as *Motivation through monitoring,* and a prominent reason to find the self-test useful. Participants expressed, that by following one’s results, it could encourage people to start, or continue with fall prevention exercises to improve balance. None of the other studies, using the UX Honeycomb model as a framework, related to motivation. 

Further, within the part Use features of more practical matters evolved. One example from *Essential with clear instructions*, was the importance of giving instructions with short, specific word like up/down, in order for the user to understand and perform the test correctly. Moreover, during the design of the user interface, considerations about how to *Adapt for vision and hearing impairment* were prominent in the process with the older adults. *Facilitate for different levels of cognition* was addressed and re-addressed together with the participants in order to reach feasible solutions. Additionally, the aspect of *Organize information logically and simply* to make the user interface easy to use (logic, details, colors) was a central aspect in the design process. Independent of what age-group the user interface design is for, the design of the interface is important to facilitate use of the application. Overall, Use was the part where most suggestions came up during the co-creation sessions. The other three studies, using the UX Honeycomb model, reported resembling issues featuring use. Parents expressed the importance of a balance between audio, video, and written content [23]. Patients with glaucoma conveyed the necessity of limiting the content [22]. In the Decision Boxes study, the need for synthesized and simplified information was expressed, especially “Having a good impression first” including Simplicity, Choice of color, Avoid excessive information, and Individuals previous levels of knowledge [20].

Independent of the UX Honeycomb model, our study likewise relates to other studies concerning older adults’ use of technology. For example, a user-test study of three different smartphone applications, showed the importance of appropriate use of colors, easy navigation, and enhanced and simplified data visualizations [25]. Some guidelines were created from this study to improve usability for older users. Although our co-creation process was completed before the publication of these guidelines, they agree with the principles we applied during our development. Further, a study, where older adults with heart failure used mHealth-based interventions, described facilitators like willingness to learn, ease of use, and the presence of useful features [41]. The participants in our study expressed similar opinions during the sessions, for example, interest in learning new things, that the interface was easy to use, and that they appreciated adaptations for reduced vision, hearing, or cognition. Moreover, a review of different eHealth interventions for seniors (60+) aging in their own homes, reported on factors influencing the acceptance of technology use, for example, positive experiences and benefits of technology, the importance of reducing problems with technology, and the influence of social networks for support [27]. Our participants discussed similar elements of acceptance, for example, they thought MyBalance would be useful by showing changes in test performance, and they did not want any hassle with technology. The role of a spouse or close friend was mentioned as important for sharing good results with, but not in terms of getting help with the technology. This could indicate that the handling of the application was uncomplicated.

A similar study has been ongoing in parallel to ours, within the European project PreventIT, however in this study co-creation was not used in the design process [42]. During their user-testing (with three iterations) they received comparable suggestions for developments, as we gained during the co-creation process. After their first user-test, video instructions were developed. After the second, the instructions were changed as confusing signals caused puzzlement, and the placement of the smartphone was often wrong. Their third user-test was done in participants’ homes, where additional challenges for standardized tests emerged. The results from our two studies correspond and therefore strengthen our gained knowledge, such as the positive effect of providing video instructions, and the need for clear instructions.

Some methodological reflections for this co-creation study will be addressed below, including both strengths and limitations. Since abundant suggestions came up throughout the sessions, we found the number of participants appropriate, and the purposive sample selection successful. The participants were committed to the assignment, and became engaged in the group, generously sharing their opinions during sessions, and a feeling of importance from taking part was noticed during this period. Non-attendance was due to being away, or illness, and on the occasion in February, snowy conditions caused only one participant to cancel their attendance. Considering the reduction in the number of participants in the final sessions, aspects of credibility and dependability must be regarded. However, the majority of participants attended throughout the 11 months and we have no indication that participation decreased due to poor interest. A summary of the session’s content was emailed to all participants after each session, something that was much appreciated. Further, the decision to include participants with some previous experience of using either a computer, tablet, or smartphone was made, as the target group for the self-test application is people that have a smartphone and are used to handling it. Participants in the study therefore also needed some experience of technology to take part in the development.

The diversity in design teams are important for the development process [28]. Our research team had a valuable mix. The engineers and physiotherapy researchers shared their knowledge and experiences during the whole process, which strengthened the development of the self-test application. As physiotherapists, the preunderstanding about the balance tests was important when developing the test instructions, though, it was also beneficial to gather participants’ perspectives on how information was received. The engineers had a key role in developing the prototype. Likewise, in the analysis phase, the combination of researchers was valued. Two of the authors were not involved in the co-creation process and had an outside perspective during the analysis. Due to this, the objectivity was upheld and confirmability was reinforced [40]. 

Further, the UX Honeycomb model seemed to be suitable to use as the categorization matrix in this study. Despite the model’s non-scientific origin it is often used in applied design [19] and fulfilled the requirements of providing a framework in our study. We therefore consider the UX Honeycomb model to be worthwhile to apply further in research regarding user experience. The model helped organize our findings from the multiple data collection, and we believe that the use of a categorization matrix improved both transferability and credibility [40]. It was satisfying to discover during the analysis how data from the sessions covered each facet of the UX model, indicating that all aspects were discussed during the sessions. In the process to make the application useful and valuable, as the highest steps indicate, the analysis showed that we had been through all the steps (findability, accessibility, usability, desirability, and credibility) along the way. By thoroughly describing the conditions during the co-creation sessions as well as the analysis process and framework, we tried to facilitate transferability of our results to other similar developments of applications for older adults [40]. To provide good user experience various guidelines for application development exist. Even though no specific guideline was systematically followed at this early stage of the development process, many aspects were still implemented. Our focus was to accomplish a simplistic design that even inexperienced smartphone users would find intuitive. In further development of the MyBalance app specific guidelines will be considered. Since our co-creation process was guided by the direct response from older participants, we hope our results could augment future design processes aiming to develop self-test applications for older adults.

Further research is needed before the MyBalance app can be released. A proper user-test study to test the current prototype, refined during our co-creation sessions, is needed. Additionally, future work of validity and reliability is required to confirm the application’s algorithms, and how the user handles the self-test. Moreover, the desire among some participants to be able to compare their results with normal values requires a large data collection and the MyBalance app might be able to provide this in the future. 

There is a gap to fill in health services regarding the prevention of falls and new methods to prevent falls are needed as resources are limited. Self-managed fall prevention exercises could be a step forward to help older adults reduce the number of falls. A self-test might help improve motivation for doing fall preventive exercises and evaluate the results, thereby increase adherence in such interventions.

## 5. Conclusions

The user experience aspect was central, in this 11 month long co-creation process together with 10 older adults, when designing the self-test application MyBalance. The UX Honeycomb model served well to organize the participants’ input in order to understand the experienced value of the smartphone application. All aspects of the model for user experience were covered during the design development. The results from the analysis indicate that the co-creation sessions generated the most novel input in relation to how participants felt regarding the application and their desires for different features in the application’s design. Further, the credibility and usefulness were also discussed among participants, and together with the more practical matters, several suggestions for the design were provided. Their wish to achieve knowledge and understand why, was evident during the co-creation process. Additionally, the appropriate level of clear information was important, neither too much nor too little. Furthermore, they also expected the MyBalance self-test to be a useful application to test balance and leg strength and in addition, to help motivate performance of exercises to prevent falls. In conclusion, the Optimized Honeycomb model for user experience was found to be a useful tool for analyzing and describing the participants’ preferences when developing a new self-test application for older adults.
